# A deletion in the *Ctns* gene causes renal tubular dysfunction and cystine accumulation in LEA/Tohm rats

**DOI:** 10.1007/s00335-018-9790-3

**Published:** 2018-12-27

**Authors:** Yukiko Shimizu, Rieko Yanobu-Takanashi, Kenta Nakano, Kenji Hamase, Toshiaki Shimizu, Tadashi Okamura

**Affiliations:** 1 Department of Laboratory Animal Medicine, Research Institute, National Center for Global Health and Medicine (NCGM), 1-21-1 Toyama, Shinjyuku-ku, Tokyo, 162-8655 Japan; 2 Department of Pediatrics and Adolescent Medicine, Juntendo University Graduate School of Medicine, 2-1-1 Hongo, Bunkyo-ku, Tokyo, 113-8421 Japan; 3 Laboratory of Laboratory Animal Science and Medicine, School of Veterinary Medicine, Kitasato University, Higashi 23-35-1, Towada, Aomori 034-8628 Japan; 4 Graduate School of Pharmaceutical Sciences, Kyushu University, 3-1-1 Maidashi, Higashi-ku, Fukuoka, 812-8582 Japan; 5 Section of Animal Models, Department of Infections Diseases, Research Institute, National Center for Global Health and Medicine (NCGM), 1-21-1 Toyama, Shinjyuku-ku, Tokyo, 162-8655 Japan

## Abstract

The Long-Evans Agouti (LEA/Tohm) rat has recently been established as a new rat model of type 2 diabetes. The onset of diabetes mellitus was observed only in male LEA/Tohm rats; however, urinary glucose appeared before the onset of diabetes. To clarify the genetic basis of urinary glucose, we performed genetic linkage analysis using (BN × LEA) F_2_ intercross progeny. A recessively acting locus responsible for urinary glucose excretion (*ugl*) was mapped to a 7.9 Mb region of chromosome 10, which contains the cystinosin (*Ctns*) gene. The *Ctns* gene encodes the cystine transporter, which transports cystine out of the lysosome and is responsible for nephropathic cystinosis in humans. Sequence analysis identified a 13-bp deletion in the *Ctns* gene, leading to a truncated and loss-of-function protein, which cause cystine accumulation in various tissues. We also developed a novel congenic rat strain harboring the *Ctns*^*ugl*^ mutation on the F344 genetic background. Phenotypic analysis of F344-*Ctns*^*ugl*^ rats indicated that the incidence of urinary glucose was 100% in both males and females at around 40 weeks of age, and marked cystine accumulation was observed in the tissues, as well as remarkable renal lesions and cystine crystals in the lysosomes of the renal cortex. Furthermore, treatment with cysteamine depleted the cystine contents in F344-*Ctns*^*ugl*^ rat embryonic fibroblasts. These results indicated that the F344-*Ctns*^*ugl*^ rat provides a novel rat model of cystinosis, which allows not only a better understanding of the pathogenesis and pathophysiology of cystinosis but will also contribute to the development of new therapies.

**Electronic supplementary material:**

The online version of this article (10.1007/s00335-018-9790-3) contains supplementary material, which is available to authorized users.

## Introduction

Cystinosis is a rare progressive lysosomal storage disorder characterized by the accumulation of cystine in lysosomes throughout every cell in the body. This results in various clinical characteristics such as Fanconi syndrome, poor growth, photophobia, hypothyroidism, end-stage renal disease (ESRD), hypothyroidism, hypogonadism, muscle weakness, swallowing difficulties, and pulmonary dysfunction. In untreated individuals, glomerular function gradually deteriorates, resulting in renal failure at approximately 10 years of age (Gahl et al. 2002; Gahl 1986). Cystinosis is an autosomal recessive disorder caused by mutations that disable the cystine transporter called cystinosin (CTNS) (Town et al. 1998; Touchman et al. 2000). The *CTNS* gene encodes a seven-transmembrane domain-containing, cystine/H^+^ symporter lysosomal protein, which has an indispensable role in the efflux of cystine from the lysosome (Kalatzis et al. 2001). In patients with cystinosis, cystine (which is the oxidized dimer form of cysteine) accumulates continuously in the lysosome owing to the defective CTNS, resulting in the formation of cystine crystals in every cell in the body, leading to multi-organ damage. It is estimated that the frequency of cystinosis is 1 in 100,000–200,000 live births (Elmonem et al. 2016; Gahl et al. 2002); however, the incidence in East Asia is much lower (Chuang et al. 2012; Higashi et al. 2017; Yang et al. 2015) because the disease is often undiagnosed or misdiagnosed.

Cystinosis is one of the few rare diseases that have specific treatments. Oral cysteamine treatment, which depletes lysosomal cystine in all body cells and tissues, improves the prognosis of cystinosis by delaying the progression to ESRD (Brodin-Sartorius et al. 2012). Oral cysteamine therapy is effective in extending life expectancy and patient survival, but cannot cure the disease completely (Cherqui 2012; Gahl et al. 2007). However, new therapeutic approaches such as bone marrow and hematopoietic stem cell transplants (Gaide Chevronnay et al. 2016; Syres et al. 2009; Yeagy et al. 2011) are expected to offer a cure for cystinosis. *Ctns* knockout mice are thus useful for the development of new therapies, and to reveal the pathogenesis of cystinosis (Nevo et al. 2010; Prencipe et al. 2014).

Large amounts of urine with a strong odor, a possible indicator of diabetes mellitus, were frequently observed, and urinary glucose was detected in both male and female LEA/Tohm rats. We have previously reported a novel rat model of non-obese type 2 diabetes, the Long-Evans Agouti (LEA/Tohm) rat, which is characterized by a spontaneous insulin secretion-deficient diabetes model (Okamura et al. 2013). Spontaneous diabetes mellitus, as determined by oral glucose tolerance, was observed only in male LEA/Tohm rats, with the incidence of approximately 90% at 14 months of age, whereas none of the females developed diabetes. However, renal glucosuria appeared before the onset of diabetes in male rats and was present in all males and females at 9 months of age. Furthermore, the deterioration of renal tubular cells was evident in the proximal tubules at 12 months of age; in contrast, no proliferation of glomerulosclerosis or mesangial cells characteristic of diabetic nephropathy was observed, suggesting that renal glucosuria was not associated with the onset of diabetes. Therefore, we speculated that the renal tubular epithelial cells failed to reabsorb glucose, resulting in the development of renal glucosuria in LEA/Tohm rats and that diabetes mellitus observed in males is not correlated with renal glucosuria. In the present study, we identified the gene mutation responsible for glucosuria in LEA/Tohm rats (designated *Ctns*^*ugl*^) using a genetic association study and a candidate gene approach. We also established a novel congenic strain harboring the *Ctns*^*ugl*^ mutation derived from the LEA/Tohm rat on a F344 standard rat strain, with the goal of establishing a novel rat model to be used for the further study of cystinosis.

## Materials and methods

### Animals

The LEA/Thom rats were obtained from the Institute for Animal Experimentation, Tohoku University Graduate School of Medicine, and were maintained at the Research Institute at the National Center for Global Health and Medicine (NCGM). BN/ssNSlc and F344/NSlc rats were purchased from Japan SLC (Hamamatsu, Japan). All rats were housed in an air-conditioned animal room at 23 ± 2 °C and relative humidity of 40–60% under specific-pathogen-free (SPF) conditions, with a 12-h light/dark cycle (8:00–20:00/20:00–8:00). All rats were fed a standard rodent CE-2 diet (CLEA Japan, Tokyo, Japan) and had *ad libitum* access to water.

### Genetic mapping

For genetic mapping of the *ugl* mutant locus, F_1_ progeny were generated by crossing LEA/Tohm rats with BN rats, which were then used to produce the F_2_ intercross progeny. Genomic DNA samples from 47 F_2_ intercross progeny were genotyped using polymorphic 201 microsatellite markers (hereinafter referred to as SSLP markers, Supplemental Table S1). Genotyping was carried out via PCR and 4% agarose electrophoresis. A total of 196 F_2_ intercross progeny were genotyped for the *ugl* locus as per the presence or absence of urine glucose. Eleven SSLP markers (including an additional five markers) located on rat chromosome 10 were used for fine mapping (Supplemental Table S1). The association between the *ugl* locus and each SSLP marker were individually evaluated via *χ*^2^ test using JMP7 statistics software (SAS Institute Japan, Tokyo, Japan) (Masuyama et al. 2003).

### Identification of the *Ctns* mutation

The total RNAs were extracted from the kidneys of LEA/Tohm and BN rats using an ISOGEN RNA extraction kit (Nippon Gene, Tokyo, Japan) according to the manufacturer’s instructions. The total RNA was made to react with random hexamer primers and ReverTra Ace reverse transcriptase (Toyobo, Osaka, Japan). PCR analyses were performed on cDNA using primer pairs (5′-AGTGATAGGTGGAGAGGACAGAA-3′; 5′-ACCAGGCCATGAAGTAGA-3′ and 5′-GGTCTCTCTGCTCCTCCC-3′; 5′-TCGTACCCTGGTTTCTTTCTGTA-3′) for amplifying the entire coding regions of the *Ctns* gene. Sequence analysis was performed with an ABI PRISM 3100 Genetic Analyzer (Applied Biosystems, Foster City, CA, USA) and GENETYX-MAC Ver .13 (GENETYX, Tokyo, Japan).

### Development of congenic strain

We created a congenic strain using marker-assisted selection protocols (Markel et al. 1997), the so-called “speed congenic” methods, for reducing the number of backcross generations needed to establish the congenic strain. The individual with the closest F344 strain type was selected from each generation using microsatellite genotyping, which was carried out via PCR with SSLP markers (Supplemental Table S2). To introgress the *Ctns*^*ugl*^ mutation from LEA/Tohm into the inbred F344 strain (F344-*Ctns*^*ugl*^), the F_1_ (F344 × LEA/Tohm) progeny heterozygous for *Ctns*^*ugl*^ mutation were backcrossed to F344 rats for seven generations, and homozygous F344-*Ctns*^*ugl*^ rats were subsequently produced and maintained by sib-mating. F344-*Ctns*^*ugl*^ rats deposited to the National BioResource Project-Rat (NBRP-Rat).

### RT-qPCR analysis

RT-qPCR analyses were performed as previously described, with minor modifications (Nakano et al. 2018). Briefly, total RNAs from the kidneys and livers of 14-week-old male BN and LEA/Tohm rats were extracted as described above, and cDNA was prepared with ReverTra Ace (Toyobo). For quantification, *Ctns* gene-specific primers (5′-TGGATCTACTTCATGGCCTGGTC-3′ and 5′-TAAGCTGAAGAAGGCGTCGTT-3′) were used with THUNDERBIRD SYBR qPCR Mix (Toyobo) in a 7900HT Fast Real-Time PCR System (Applied Biosystems). The expression level of the *Ctns* gene was normalized to the level of *Actb* mRNA amplified by specific primers (5′-CGCATCCTCTTCCTCCCT-3′ and 5′-CAGACAGCACTGTGTTGGCAT-3′). Each experiment was performed in three biological replicates.

### Measurement of urine glucose

Urine samples from 16-h-fasted rats were collected by gentle manual compression of the abdomen. Urine glucose levels were measured semiquantitatively using uropaper AG2 (Eiken, Tokyo, Japan). Glucosuria is defined as a urine glucose level of 50 mg/dL or higher.

### Measurement of tissue cystine contents

Rat tissues were homogenized in 500 µL of 0.9% saline and were then deproteinized in 3 mL of 5% trichloroacetic acid solution with an internal standard (l-norvaline, Wako Pure Chemical Industries, Ltd., Osaka, Japan). After adding an equivalent volume of hexane, the homogenates were mixed and centrifuged for 15 min at 1600×*g* at room temperature and the aqueous portion (lower phase) was collected. For sensitive detection, cystine molecules were derivatized with 4-fluoro-7-nitro-2,1,3-benzoxadiazole (NBD-F) (Hamase 2007). To 5 µL of the aqueous solution, 5 µL of H_2_O, 10 µL of 400 mM Na-borate buffer (pH 8.0), and 5 µL of 40 mM NBD-F (Tokyo Kasei Kogyo, Tokyo, Japan) in acetonitrile solution (Nacalai Tesque, Kyoto, Japan) were added, and the solution was heated at 60 °C for 2 min. After adding 75 µL of 2% trifluoroacetic acid (TFA) to terminate the reaction, an aliquot of this reaction mixture (2 µL) was subjected to high-performance liquid chromatography (HPLC, Shimadzu Corporation, Kyoto, Japan) with Nucleonavi column (1.0 mm I.D × 250 mm; Shiseido, Tokyo, Japan), to quantify the cystine-NBD derivatives. The mobile phase was 22.5% v/v acetonitrile and 0.05% v/v TFA in H_2_O at the flow rate of 100 µL/min, and fluorescence detection was carried out at 530 nm with excitation at 470 nm.

### Histopathological analysis

Rats were anesthetized using sevoflurane and were perfused intracardially with PBS followed by 10% neutral buffered formalin (Nacalai Tesque). The kidneys were removed and embedded in paraffin according to routine procedures. Paraffin sections were sliced at a thickness of 3 µm and stained with hematoxylin and eosin (HE). Masson’s trichrome staining was also conducted to assess fibrosis in the kidney. The stained sections were captured using optical microscopy (BX-51; Olympus, Tokyo, Japan).

For electron microscopy, the kidneys were fixed in 2.5% glutaraldehyde/PBS solution for 2 h at room temperature. Fixed tissues were washed with Milli Q water three times and stained en bloc with 0.5% uranyl acetate for 2 h, dehydrated in an ethanol series, and then embedded in Quetol 812 (Nisshin EM, Tokyo, Japan). The blocks were trimmed to an area of approximately 0.5 mm^2^ and serially sectioned at a thickness of 100 nm with an ultramicrotome (Leica EM UC7, Leica, Vienna, Austria). The thin sections were then examined using an electron microscope (JEM-1400, JEOL, Tokyo, Japan).

### Preparation of rat embryonic fibroblast cells and drug assay

Pregnant F344-*Ctns*^*ugl/ugl*^ rats at 15 d.p.c (day post-coitum) were euthanized via sevoflurane overdose. The uterine horns were dissected out, briefly rinsed in 70% (v/v) ethanol, and washed with PBS. Each embryo was separated from its placenta and embryonic sac, and the head and red organs were removed from isolated embryos. The remaining bodies were washed with PBS and minced with scissors until they are possible to pipette. The tissues were transferred into 0.1 mM trypsin/1 mM EDTA solution (Nacalai Tesque), and incubated at 37 °C with mixing by inversion for 20 min. The suspension was filtered using a cell strainer (BD biosciences, Bedford, MA, USA), followed by centrifugation for 5 min at 160×*g*. The pellets were dissociated by gentle pipetting in REF culture medium (DMEM containing 10% fetal bovine serum). The P0 (passage 0) fibroblasts were grown in REF medium until P8 in 5% CO_2_:95% (v/v) air, and used for drug analyses. The cells were incubated in REF medium containing cysteamine, bucillamine (Wako Pure Chemical Industries, Ltd.), or captopril (Sigma-Aldrich, St. Louis, MO, USA) for 24 h. The cystine contents of the drug-treated cells were determined via HPLC as described above.

### Statistics

Data were presented as mean ± standard error of the mean (SEM). Data were analyzed using Student’s *t* test. Correction of multiple comparisons was calculated using Bonferroni correction. Statistical analysis was performed using Excel Statistic and JMP (ver.7.0.1) software. A *P* value < 0.05 was considered statistically significant.

## Results

### Linkage analysis and identification of the *ugl* mutation

**Fig. 1:**
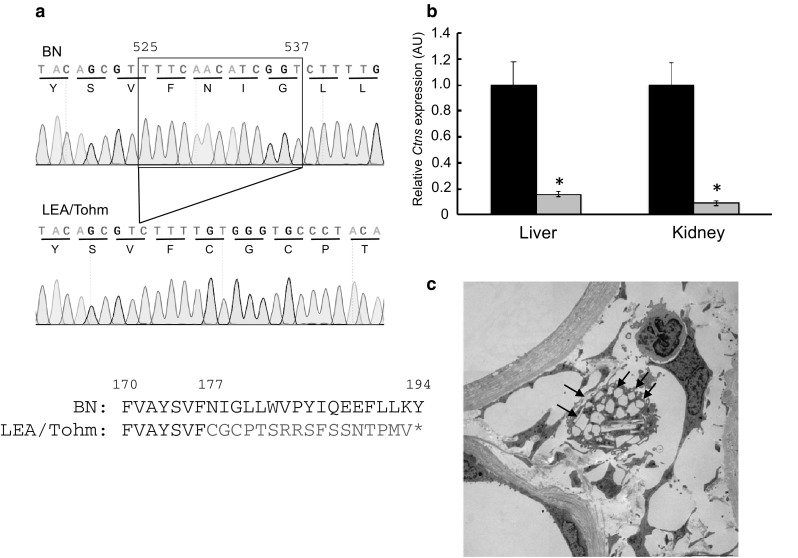
Mutation analysis of *Ctns* in LEA/Tohm rats. **a** Partial electropherogram of Sanger sequencing of the *Ctns* cDNA from BN (+/+, upper panel) and LEA/Tohm (*ugl/ugl*, middle panel) rats. The reading frame is underlined. A 13-bp deletion (c.525_537del) causes a frame shift mutation at codon 177, with subsequent termination after additional 17 codons (bottom panel). The stop codon is shown as an asterisk. **b** RT-qPCR analysis of the *Ctns* gene in LEA/Tohm liver and kidneys at 14 weeks of age. The values were arbitrary units after normalization against *Actb*. Data were analyzed using Student’s *t* test (*P < 0.01). BN (closed bars; *n* = 3) and LEA/Tohm (gray bars; *n* = 3) rat. Each experiment was performed in three biological replicates. **c** A representative transmission electron microscopy (TEM) image of the LEA/Tohm kidney cortex at 52 weeks of age. Arrows indicate cystine crystals in macrophages of the renal interstitium. Magnification ×2500

Though only male LEA/Tohm rats developed diabetes mellitus with the incidence of 90% at 14 months of age, the urinary glucose was positive before the onset of diabetes at 9 months (approximately 40 weeks) of age (Okamura et al. 2013). To determine the mode of inheritance of renal glycosuria in LEA/Tohm rats, we generated (BN × LEA/Tohm) F_2_ intercross progeny. Of the 196 male offspring, 52 (26.5%) and 144 (73.5%) individuals at 52 weeks of age were positive and negative for urinary glucose, respectively, indicating that renal glycosuria in LEA/Tohm rats is inherited in a recessive fashion. Next, we genotyped DNA samples from the 47 male F_2_ intercross progeny using 201 SSLP markers (Supplemental Table S1) on rat chromosomes 1–20 and X to determine the urinary glucose excretion (*ugl*) locus. Using *χ*^2^ analysis, we detected significant association of the *ugl* locus with SSLP markers on chromosomes 2, 8, 10, and 16 and mapped the *ugl* locus to an interval of approximately 45 Mb between *D10Rat34* (*P* = 2.2E−5) and *D10Rat151* (*P* = 5.85E−7) around *D10Mgh6* (*P* = 7.1E−12) (Table 1). By using the male 196 F_2_ intercross progeny and examining an additional five SSLP markers on chromosome 10 (Supplemental Table S1), the haplotype analysis revealed that the *ugl* locus was located within an interval of 7.9 Mb between *D10Rat77* (*P* = 3.16E−41) and *D10Mgh6* (*P* = 1.96E−43) (Table 2, Supplemental Fig. S1). No recombination could be seen between the *ugl, D10Rat241* and *D10Rat80* (*P* = 1.56E−44). This region contains 132 protein-coding genes, including the *ugl* candidate region [The Rat Genome Database (RGD) (https://rgd.mcw.edu)]. Given the information regarding the candidate genes for this locus based on RGD, *Ctns* was the strongest candidate gene for the *ugl* locus. By analyzing the sequence of *Ctns*, which consists of 10 coding exons and encodes 367 amino acids, we identified a 13-bp deletion at position 525 (c.525_537del) in exon 7 of *Ctns* in LEA/Tohm rats (Fig. 1a), causing a frame shift mutation at codon 177 with subsequent termination after an additional 17 codons. We designed genotyping PCR primers to detect the 13-bp deletion in the *Ctns* gene, and examined the variation in exon 7 of *Ctns* among the LEA substrains. The 13-bp deletion was observed in LEA/Tohm and LEA/Hkm, but not in LEA/Tj (Nakajima et al. 1994) and LEA/Hok rats (Agui et al. 2001) (Supplemental Fig. S2).

**Table 1:** SSLP markers showing significant associations between genotype and urine glucose positivity on 47 F_2_ intercross progeny

Chr.	Locus name	Position (Mb)^a^	Urine glucose-negative^b^		Urine glucose-positive^c^		*χ* ^2^	*P* value
			BN/BN or BN/LEA	LEA/LEA	BN/BN or BN/LEA	LEA/LEA		
2	*D2Rat112*	249.3	27	8	5	7	5.17	0.02
8	*D8Rat47*	39.3	30	5	7	5	4.00	0.05
10	*D10Rat121*	16.8	27	8	5	7	5.18	0.02
	*D10Rat181*	17.7	28	7	5	7	6.28	0.01
	*D10Rat42*	21.7	28	7	5	7	6.28	0.01
	*D10Rat34*	32.5	31	4	3	9	18.1	2.20E−05
	*D10Mgh6*	64.6	35	0	0	12	47.0	7.10E−12
	*D10Rat151*	77.5	32	3	2	10	25.0	5.85E−07
	*D10Rat122*	91.3	29	6	4	8	10.5	1.20E−03
16	*D16Rat34*	73.0	25	10	12	0	4.36	0.04
	*D16Rat37*	75.8	23	12	12	0	5.52	0.02

*χ*^2^ test *P* value lower than 0.05 are shown. Whole genome scan was performed in 47 F_2_ progeny using 201 SSLP markers as shown in Supplemental Table S1

^a^*Rattus norvegicus* (Norway rat) genome assembly Rnor_6.0

^b^F_2_ rats having urine glucose lower than 50 mg/dL under fasting conditions

^c^F_2_ rats having urine glucose higher than 50 mg/dL under fasting conditions

**Table 2:** *χ*^2^ test for association between genotype on rat chromosome 10 and urine glucose positivity using 196 F_2_ intercross progeny

Locus name	Position (Mb)^a^	Urine glucose-negative^b^		Urine glucose-positive^c^		*χ* ^2^	*P* value
		BN/BN or BN/LEA	LEA/LEA	BN/BN or BN/LEA	LEA/LEA		
*D10Rat95*	6.1	110	34	36	16	1.03	0.31
*D10Rat42*	21.7	122	22	27	25	22.5	2.05E−06
*D10Rat34*	32.5	131	13	16	36	73.8	8.43E−18
*D10Rat223*	42.8	134	10	10	42	106.8	4.87E−25
*D10Rat77*	56.7	143	1	2	50	180.90	3.16E−41
*D10Rat241*	59.7	144	0	0	52	196.0	1.56E−44
*ugl, Ctns*	59.75–59.77						
*D10Rat80*	61.3	144	0	0	52	196.0	1.56E−44
*D10Mgh6*	64.6	143	1	0	52	191.0	1.96E−43
*D10Rat133*	64.8	141	3	1	51	176.4	3.01E−40
*D10Rat151*	77.5	136	8	13	39	101.10	8.89E−24
*D10Rat7*	104.3	118	26	36	16	3.67	0.06

^a^*Rattus norvegicus* (Norway rat) genome assembly Rnor_6.0

^b^F_2_ rats having urine glucose lower than 50 mg/dL under fasting conditions

^c^F_2_ rats having urine glucose higher than 50 mg/dL under fasting conditions

Next, we examined the mRNA expression of the *Ctns* gene in the liver and kidneys from BN and LEA/Tohm rats via quantitative RT-PCR. The relative abundance of *Ctns* transcripts from LEA/Tohm rats is markedly lower in the liver (16%) and kidney (10%) (Fig. 1b), which may be caused by nonsense-mediated mRNA decay (NMD), resulting in systemic cystine accumulation in all tissues. Therefore, the intracellular cystine content of different organs from LEA/Tohm and BN rats was assayed via HPLC. Cystine content was significantly increased in the liver, eyes, heart and spleen of LEA/Tohm rats at 8–14 weeks of age in comparison to that of BN rats (Table 3). At 52 weeks of age, cystine accumulation was clearly observed in all organs tested, with the highest levels in the spleen (430-fold) and the lowest in the brain (1.8-fold) compared with age-matched BN rats. An increase of 12.3-fold was seen in the liver, 3.9-fold in the kidneys, 83-fold in the heart, 9.2-fold in the lungs, 36-fold in the muscles, and 90-fold in the eyes (Table 3), and these accumulations increased with age. Cystine crystal structures, which are one of the typical pathological findings in cystinosis patients (Gahl et al. 2002) and mouse models of cystinosis (Cherqui 2002; Nevo et al. 2010), were present in the kidney cortex (Fig. 1c). These results indicate that renal glycosuria in the LEA/Tohm rat was caused by a deletion of 13-bp in *Ctns*.

**Table 3:** Intracellular cystine levels in various tissues of LEA/Tohm rats

	Liver	Kidney	Heart	Lung	Muscle	Brain	Eye	Spleen
8–14 weeks								
BN (*n* = 5)	0.12 ± 0.02	1.25 ± 0.07	0.21 ± 0.03	0.34 ± 0.02	0.02 ± 0.00	0.26 ± 0.02	0.04 ± 0.00	0.08 ± 0.01
LEA (*n* = 5)	0.34 ± 0.07*	1.50 ± 0.26	0.47 ± 0.10*	0.39 ± 0.03	0.10 ± 0.03	0.28 ± 0.05	0.12 ± 0.03*	5.18 ± 2.48*
52 weeks								
BN (*n* = 3)	0.10 ± 0.03	1.45 ± 0.10	0.07 ± 0.03	0.29 ± 0.03	0.01 ± 0.00	0.13 ± 0.05	0.02 ± 0.00	0.08 ± 0.01
LEA (*n* = 3)	1.23 ± 0.02**	5.60 ± 1.67*	5.81 ± 0.45**	2.66 ± 0.18**	0.36 ± 0.06**	0.23 ± 0.05	1.79 ± 0.22**	34.34 ± 0.87**

Tissue cystine contents were measured as micromoles of half-cystine per gram of wet tissue, mean ± SEM

*P < 0.05; **P < 0.01 versus age-matched control BN rats

### Quantitative Trait Loci (QTL) analysis for kidney function

To examine whether glucosuria was correlated with renal function in LEA/Tohm rats, we performed QTL analysis for serum creatinine using the same F_2_ intercross progeny as mentioned above (Supplemental Fig. S3). We identified one significant QTL peak on chromosome 10 at *D10Mgh6* (64.6 Mb) with a likelihood ratio statistic (LRS) score of 64.1, and one suggestive QTL peak on chromosome 12 at *D12Rat23* (27.2 Mb) with an LRS score of 12.8 (Supplemental Fig. S4a). *D10Mgh6* is located close to the *ugl* locus, which is responsible for urinary glucose excretion (Supplemental Fig. S4b) strongly indicating a correlation between the two phenotypes.

### Development of a novel rat model for cystinosis

**Fig. 2:**
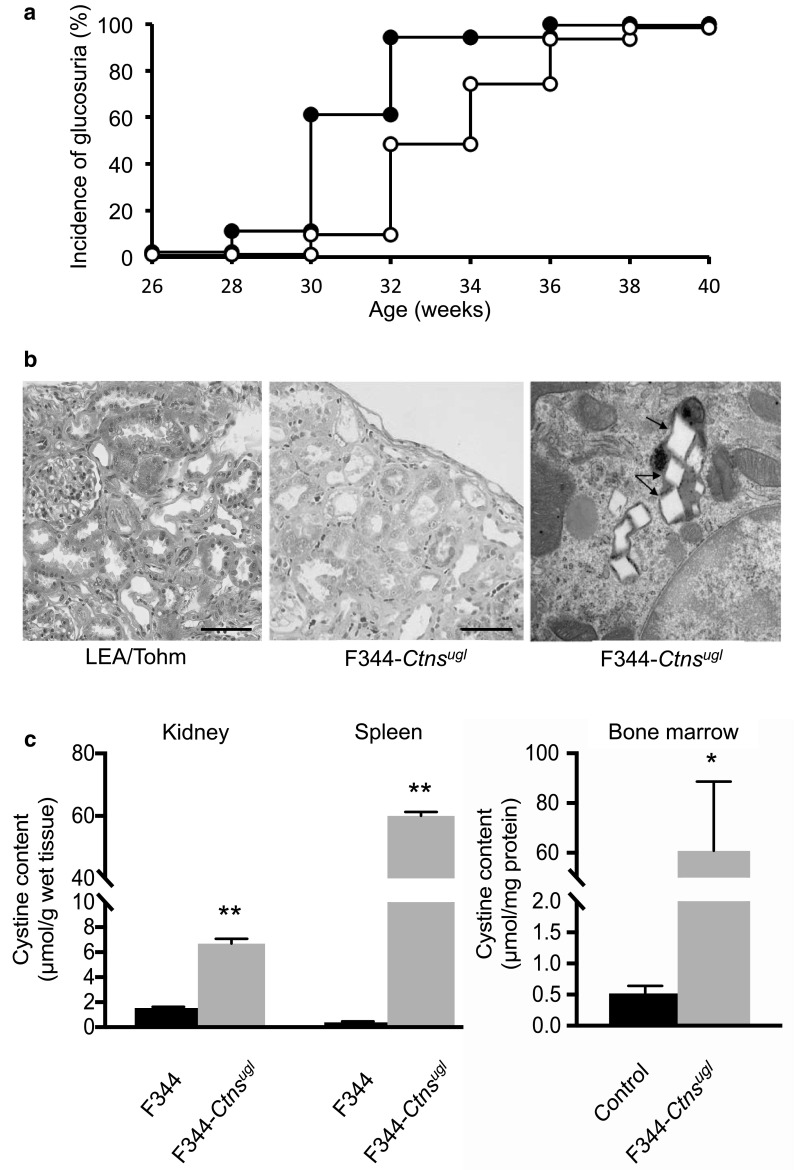
Phenotypic analysis of F344-*Ctns*^*ugl*^ rats. **a** The cumulative incidence of glucosuria in F344-*Ctns*^*ugl*^ male (closed circle; *n* = 18) and female (open circle; *n* = 31) rats. Glucosuria is defined as urine glucose levels of 50 mg/dL or higher under fasting conditions. **b** Renal histological section of male LEA/Tohm at 48 weeks of age (left panel) and male F344-*Ctns*^*ugl*^ rats at 52 weeks of age (middle panel) stained with Masson’s Trichrome. Scale bar = 100 µm. Right panel: Representative TEM image of male F344-*Ctns*^*ugl*^ kidney cortex at 52 weeks of age. Arrows indicate cystine crystals in lysosomes. Magnification ×25,000. **c** Cystine contents of kidney and spleen (left panel) at 38–42 weeks of age, and of bone marrow (right panel) at 40 weeks of age in male F344 (*n* = 3; black bars) rats and male F344-*Ctns*^*ugl*^ (*n* = 3; gray bars) rats. *P < 0.05, **P < 0.01

The LEA rats were established from an outbred colony of Long-Evans rats, and were sometimes used as a control strain for the Long-Evans Cinnamon (LEC) rats, a rat model for Wilson’s disease, since they do not have the *Atp7b* mutation (Sasaki et al. 1994). However, LEA rats carry various other mutations such as impaired glucose tolerance (Okamura et al. 2013), X-ray hypersensitivity (Agui et al. 2001), and lack of d-amino acid oxidase activity (Konno et al. 2009), and are, therefore, not necessarily suitable as a cystinosis model. We established a congenic rat line in which the *Ctns*^*ugl*^ mutation was introduced into F344 rats using speed congenic methods (Supplemental Table S2). Glucosuria appeared at 28 weeks of age in the homozygous male F344-*Ctns*^*ugl*^ rats, and was present in 100% of males at 36 weeks of age. In the homozygous female rats, glucosuria appeared at 30 weeks of age and was present in 100% of females at 38 weeks of age (Fig. 2a). As seen in the kidneys of LEA/Tohm rats (Okamura et al. 2013), pathological analysis of F344-*Ctns*^*ugl*/*ugl*^ kidneys at 52 weeks of age revealed markedly expanded tubular lumen in the cortex as well as proximal tubular atrophy, flattened epithelium, and basement membrane thickening associated with the disappearance of the epithelial cell layer (Fig. 2b). Electron microscopy of the kidney cortex showed that a large number of cystine crystal structures were also present in intracellular lysosomes (Fig. 2b), as also seen in LEA/Tohm kidneys (Fig. 1c). Cystine content was markedly increased in all F344-*Ctns*^*ugl*/*ugl*^ rat tissues tested at 52 weeks of age. An increase of 4.3-fold was seen in the kidney, 154-fold in the spleen, and 117-fold in the bone marrow (Fig. 2c). Despite this, the F344-*Ctns*^*ugl*/*ugl*^ rat demonstrated normal growth and fertility. In addition, F344-*Ctns*^*ugl*/*ugl*^ rats had normal glycemic response during oral glucose tolerance test (Supplemental Fig. S5), indicating that *ugl* locus on chromosome 10 is not associated with impaired glucose tolerance.

**Fig. 3:**
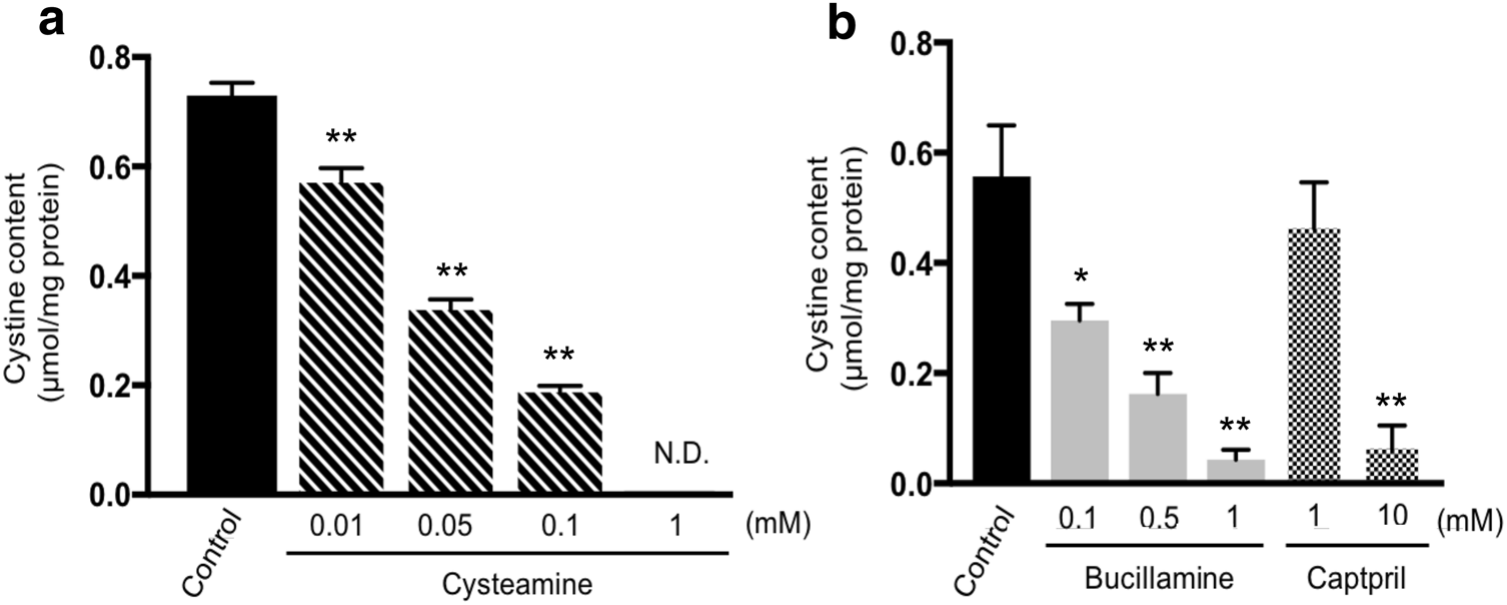
Cystine contents of F344-*Ctns*^*ugl*^ REFs treated with drugs containing thiol groups: **a** cysteamine (hatched bars), **b** bucillamine (gray bars), and captopril (dotted bars). REFs were incubated in culture medium containing each drug for 24 h. Control cells (black bar) were cultured under the same conditions, with the addition of REF medium in place of the drug. *P < 0.05, **P < 0.01 versus control. N.D. signifies “not detected”

Furthermore, we established rat embryonic fibroblasts (REFs) from F344-*Ctns*^*ugl*/*ugl*^ rats to test the effectiveness of cystine depletion by cysteamine, the only therapeutic drug for cystinosis, and evaluated whether the F344-*Ctns*^*ugl*/*ugl*^ rat was useful as a cystinosis model. Fibroblasts are frequently used in the study of cystinosis, especially in functional assessments (Iglesias et al. 2012; Sansanwal et al. 2010; Vitvitsky et al. 2010). The effect of drugs was evaluated based on cystine accumulation in REFs. The treatment with cysteamine resulted in a significant reduction of cystine content in a dose-dependent manner. A decrease of 22% was seen at 0.01 mM (*P* = 0.002), 53% at 0.05 mM, 74% at 0.1 mM, and > 99.8% at 1 mM cysteamine compared with the control (Fig. 3a). To show the availability of F344-*Ctns*^*ugl*/*ugl*^ REFs in evaluating candidate drugs of cystinosis, we tested bucillamine and captopril, both of which are thiol compounds, which also decreased cystine accumulation in a dose-dependent manner. At a dose of 1 mM, bucillamine (93% decrease) more effectively reduced the intracellular cystine levels than captopril (18% decrease) (Fig. 3b). These results suggest that REFs from the F344-*Ctns*^*ugl*/*ugl*^ rat may be used for evaluation of candidate drugs of cystinosis.

## Discussion

In the present study, we present several lines of evidence demonstrating that a mutation in the *Ctns* gene underlies spontaneous renal glucosuria in LEA/Tohm rats.

The mating test and linkage analysis using SSLP markers on the whole genome revealed that *ugl* was inherited as an autosomal recessive trait and mapped to the region on chromosome 10 harboring the *Ctns* gene (Table 2 and Supplemental Fig. S1). The *Ctns* gene encodes a seven-transmembrane domain-containing protein that functions to transport cystine out of lysosomes, and it has been reported that nephropathic cystinosis is caused by *CTNS* gene mutations in humans (Attard et al. 1999; Kalatzis et al. 2004; Town et al. 1998). Cystinosis is a disease that causes impaired export of cystine from the lysosome to the cytoplasm, resulting in the accumulation of cystine in all organs, which leads to multi-organ damage and associated serious renal tubule symptoms (Elmonem et al. 2016; Gahl et al. 2002). Systemic accumulation of cystine was also observed in various tissues from LEA/Tohm rats (Table 3). Although there were 132 genes in the *ugl* candidate region on chromosome 10 (Table 2, Supplemental Fig. S1), the *Ctns* gene was a strong candidate for the *ugl* mutation. The 13-bp deletion in the *Ctns* gene is predicted to cause a frameshift mutation, resulting in truncation of the peptide chain owing to a stop codon generated at amino acid position 194. In silico prediction for the mutated protein was based on the system SOSUI; classification and secondary structure prediction of membrane proteins suggested that the truncated protein yields a soluble protein (Hirokawa et al. 1998). The *Ctns* frameshift mutation is supposed to completely disrupt the function of the protein, which, because of this frameshift, is not localized to lysosomes and has lost its cystine transport function. In addition to this, the marked accumulation of cystine in REFs from the F344-*Ctns*^*ugl*/*ugl*^ rat was depleted after treatment with cysteamine, a therapeutic agent for cystinosis. We therefore conclude that the *ugl* mutation, which caused the degeneration of renal tubular epithelial cells, development of glucosuria, and cystine accumulation in LEA/Tohm rats, is the 13-bp deletion in the *Ctns* gene.

F344-*Ctns*^*ugl*/*ugl*^ rats carrying the *Ctns*^*ugl*^ mutation in the F344 genetic background exhibited renal proximal tubulopathy, and incidence of glucosuria reached 100% in both males and females at around 40 weeks of age. The onset of glucosuria in F344-*Ctns*^*ug*/*ugll*^ rats was slightly delayed compared to that in LEA/Tohm rats (Okamura et al. 2013) (Fig. 2a). Reportedly, renal dysfunction in *Ctns*^−/−^ mice was dependent on their genetic background (Nevo et al. 2010). *Ctns*^−/−^ mice on the C57BL/6 genetic background developed focal lesions affecting the proximal tubules at 6 months of age, with more extensive lesion development at 9 months, whereas the *Ctns*^−/−^ mice having FVB/N and a mixed 129 Sv × C57BL/6 genetic backgrounds present no signs of proximal tubulopathy, even at 18 months of age. The reason for these strain differences could be explained by the amount of cystine accumulation in the kidney in each congenic strain. The renal cystine levels of C57BL/6 *Ctns*^−/−^ mice were much higher than those of FVB/N and the mixed 129 Sv *Ctns*^−/−^ mice, leading to renal tubular cell damage and formation of cystine crystals. On the other hand, the elevated accumulation of cystine was clearly observed in both LEA/Tohm (3.9-fold) and F344-*Ctns*^*ugl*^ kidney (4.3-fold) compared to age-matched control strains, and cystine contents in the kidneys of these strains were similar (LEA/Tohm, 5.60 ± 1.67 vs. F344-*Ctns*^*ug*/*ugll*^, 6.68 ± 0.38 µM of half-cystine/g of wet tissue; *P* = 0.28) (Table 3; Fig. 2c). Based on these findings, it is unlikely that the early onset of glucosuria observed in LEA/Tohm rats is correlated with the amount of cystine accumulation in renal tissues. It is possible that impaired glucose tolerance (Okamura et al. 2013), which may result in glucotoxicity in the renal tubular cells, accelerated the onset of renal tubulopathy.

The cystine assay is the cornerstone in the treatment of cystinosis, both for diagnosis and therapeutic monitoring of the disease. HPLC is frequently used to detect amino acids and provides more cost-effective and easy-to-use methods compared with LC/MS/MS. Our cystine detection method using HPLC with a metal-free column successfully measured the cystine levels in various tissues obtained from control and cystinosis model rats (Table 3; Fig. 2c). Since this column does not contain metals and there was a low risk of contamination with metal impurities, we concluded that this column is more suitable for the analysis of cystine, which is the oxidized form of cysteine, and has a disulfide bond, a site of the redox reaction. However, it was difficult to measure the cystine content in the leukocytes separated from 3 mL of blood from the control strain rats using HPLC, although this content was detectable in leukocytes of F344-*Ctns*^*ugl*/*ugl*^ rats. Our cystine analysis method using HPLC is sufficient for quantifying the cystine content in cystine-accumulated tissues and leukocytes, but higher sensitivity analysis using LC/MS/MS is required for detecting the low levels of cystine in the leukocytes of healthy controls (Chabli et al. 2007; Garcia-Villoria et al. 2013).

Cysteamine, a simple aminothiol molecule, is the endogenous reducing agent and intermediate product of the taurine biosynthesis pathway. Several clinical trials have indicated that the reduction of cystine levels in cystinosis patients treated with oral cysteamine is critical to delay the disease progression and to prolong life (Brodin-Sartorius et al. 2012; Gahl et al. 2002). In cystinosis cells treated with cysteamine, the drug enters the lysosome through an unidentified transporter where it breaks the disulfide bond in cystine, leading to the formation of cysteine and cysteine–cysteamine disulfide. Cysteine and cysteine–cysteamine disulfide can leave the lysosome via cysteine and an unidentified cationic amino acid transporter, respectively, resulting in a marked reduction of cystine in the cells (Besouw et al. 2013). However, the adverse effects, which include anorexia, vomiting, unpleasant odor, and halitosis, often lead to poor compliance with oral cysteamine therapy. Additionally, cysteamine stimulates gastric acid secretion, leading to gastrointestinal ulceration when administered to rats in high doses (Selye and Szabo 1973). Therefore, the development of new therapies, such as hematopoietic stem cell transplantation (Gaide Chevronnay et al. 2016; Syres et al. 2009; Yeagy et al. 2011) and other oral medicines, is expected to improve the prognosis of cystinosis patients. We compared the effect of captopril [a specific competitive inhibitor of angiotensin I-converting enzyme (ACE)] and bucillamine (a cysteine derivative) on the reduction of intracellular cystine content using F344-Ctns^*ugl*/*ugl*^ REFs (Fig. 3). Captopril is a sulfhydryl ACE inhibitor initially used in the treatment of hypertension and heart failure, and is protective against retinopathy and nephropathy (Parving et al. 1988; UK Prospective Diabetes Study Group 1998). Bucillamine is a homolog of d-penicillamine with two thiol groups, widely used to treat rheumatoid arthritis in Japan (Sagawa et al. 2011). Treatment with captopril or bucillamine decreased intracellular cystine in a dose-dependent manner. However, treatment with captopril and bucillamine less effectively reduced the intracellular cystine levels than that with cysteamine (Fig. 3a, b). These results indicate that REFs from F344-*Ctns*^*ugl*/*ugl*^ rats are a valuable tool to evaluate and develop new cystine depleting treatments.

Oral cysteamine treatment delays disease progression and improves the life expectancy of cystinosis patients, but ultimately cannot eliminate the disease. We identified the *Ctns*^*ugl*^ mutation, which leads to cystine accumulation in various tissues and results in renal tubulopathy, and developed a novel congenic rat strain harboring the *Ctns*^*ugl*^ mutation. Although further biochemical and pathophysiological analyses of F344-*Ctns*^*ugl*^ rats are needed to evaluate their usefulness as a model of cystinosis, the evidence provided in the present study suggests that F344-*Ctns*^*ugl*^ rats will be an invaluable tool to expand our understanding of cystinosis pathogenesis, and for developing and testing novel therapeutic treatments.

## Electronic supplementary material

Below is the link to the electronic supplementary material.

Supplementary material 1 (PDF 608 KB)
