# Supplementary material for: A deletion in the Ctns gene causes renal tubular dysfunction and cystine accumulation in LEA/Tohm rats
Source: Mamm Genome. 2018 Dec 27;30(1):23–33. doi: 10.1007/s00335-018-9790-3 (PMC6397714; doi:10.1007/s00335-018-9790-3)
Supplement: Supplementary file 1 — Supplementary material 1 (PDF 608 KB) [file 335_2018_9790_MOESM1_ESM.pdf]

**List of Supporting Information**

**Appendix S1: Measurement of urinary glucose and serum creatinine…………………2**

**Appendix S2: QTL analysis…………………..…………………..…………………….2**

**Appendix S3: Genotyping of *Ctns* variants in LEA substrains…..…………………….2**

**Appendix S4: Oral glucose tolerance test……………………...…..…………………..3**

**Supplemental reference……………………………………………………………...3**

**Supplemental Table S1: SSLP markers used in genetic mapping of the rat *ugl* locus..…..4**

**Supplemental Table S2: SSLP markers used for generation of F344-*Ctns*$^{ugl}$ congenic strain**

**…..........5**

**Supplemental Fig. S1: Linkage analysis of the *ugl* locus…………………………..….........6**

**Supplemental Fig. S2: Variants of the 13-bp deletion in exon 7 of *Ctns* in LEA substrains**

**…..........7**

**Supplemental Fig. S3: Distribution of serum creatinine levels in the male 196 $F_2$ intercross progeny ......8**

**Supplemental Fig. S4: LRS score plot for QTL of serum creatinine……………………….9**

**Supplemental Fig. S5: Oral glucose tolerance test for F344-*Ctns*$^{ugl}$ rats………………….10**

**Appendix S1: Measurement of urinary glucose and serum creatinine**

Blood samples were collected from the tail veins of (LEA/Tohm × BN) F2 intercross progeny at 52 weeks of age in a fasting state. The serum creatinine concentration was measured using a Creatinine test Wako kit (Wako Pure Chemical Corporation, Japan).

**Appendix S2: QTL analysis**

For quantitative trait loci (QTL) analysis for serum creatinine, we performed a simple interval mapping method in Map Manager QTX (Manly et al. 2001) and calculated the likelihood ratio statistic (LRS) value on linkage maps with the free regression model. The significant and suggestive threshold levels for QTL were determined by permutation test in 1-cM steps for 5000 permutations using Map Manager QTX (Churchill et al. 1994; Lander et al. 1995). The same (LEA/Tohm × BN) F2 intercross progeny (a total of 196) used in genetic mapping for the *ugl* locus as described in "Material and Methods" was used for QTL analysis with polymorphic 196 SSLP markers (Supplemental Table S1).

**Appendix S3: Genotyping of *Ctns* variants in LEA substrains**

The genotyping of a 13-bp deletion in exon 7 of the *Ctns* gene was performed via PCR analysis. The PCR products were amplified with a set of primers (5'-CCCACTTTGTCCCCCTCTG-3' and 5'-TACCTGGATGTAGGGCACCCACA-3') and were separated via electrophoresis using a 4% NuSieve™ 3:1 agarose gel (Lonza, Basel, Switzerland) at 300 V for 30 min, and stained with ethidium bromide. Sanger sequencing was performed as described in "Material and Methods" to confirm the deletion in the *Ctns* gene. The genomic DNAs of LEA/ Hkm, LEA/Hok, and LEA/Tj rats were supplied by the National BioResourse Project – Rat, Kyoto University (Kyoto, Japan).

**Appendix S4: Oral glucose tolerance test**

The rats were fasted for 16 h and tail blood glucose was measured at 0, 30, 60, 90, and 120 min after the oral administration of glucose (2 g/kg body weight; Otsuka Pharmaceutical, Tokyo, Japan) by gavage.

**Supplementary reference**

Churchill RW, Doerge RW (1994) Empirical threshold values for quantitative trait mapping. Genetics 138:963-971

Lander E, Kruglyak L (1995) Genetic dissection of complex traits: guidelines for interpreting and reporting linkage results. Nat. Genet. 11: 241-247

Manly KF, Cudmore RH, Meer JM (2001) Map Manager QTX, cross-platform software for genetic mapping. Mamm. Genome 12:930-932

Supplemental Table S1  SSLP markers used in genetic mapping of the rat *ugl* locus

| *Chromosome* | SSLP Marker |
|---|---|
| 1 | *D1Rat246, D1Mgh17, D1Rat389, D1Rat403, D1Mit10, D1Mit1, D1Rat24, D1Rat27, D1Rat344, D1Rat269, D1Rat38, D1Rat271, D1Rat418, D1Rat158, D1Mgh19, D1Mit12, D1Rat139, D1Rat56, D1Rat67, D1Rat70, D1Rat169, D1Rat79, D1Rat81, D1Rat90* |
| 2 | *D2Rat189, D2Rat6, D2Mit5, D2Rat163, D2Mit17, D2Rat136, D2Rat115, D2Mit8 D2Rat40, D2Mgh12, D2Rat61, D2Rat112, D2Mit16* |
| 3 | *D3Rat52, D3Rat117, D3Rat46, D3Rat188, D3Rat130, D3Mgh6, D3Rat35, D3Rat160, D3Mit3, D3Rat145, D3Rat142, D3Mgh10* |
| 4 | *D4Rat149, D4Rat11, D4Rat157, D4Rat119, D4Rat265, D4Rat175, D4Rat182, D4Rat273, D4Rat238, D4Rat198, D4Arb40, D4Rat203, D4Rat70, D4Rat207* |
| 5 | *D5Rat126, D5Rat2, D5Rat3, D5Rat4, D5Mit10, D5Rat113, D5Rat154, D5Rat30, D5Mgh8, D5Rat99, D5Rat51* |
| 6 | *D6Mit5, D6Rat41, D6Rat148, D6Rat34, D6Rat132, D6Rat20, D6Rat14,D6Rat11, D6Rat8, D6Rat109* |
| 7 | *D7Rat113, D7Rat31, D7Rat92, D7Rat73, D7Mit5, D7Mgh6, D7Rat85, D7Rat3* |
| 8 | *D8Rat55, D8Mit5, D8Rat47, D8Rat80, D8Mgh7, D8Mgh4, D8Rat130, D8Rat90, D8Rat3* |
| 9 | *D9Rat44, D9Rat133, D9Rat129, D9Rat76, D9Mit3, D9Rat11, D9Rat84, D9Rat5, D9Rat4, D9Rat110, D9Rat153, D9Rat108, D9Rat71* |
| 10 | *D10Rat95, D10Rat121, D10Rat181, D10Rat42, D10Rat34, D10Rat223*[a], *D10Rat77*[a], *D10Rat241*[a], *D10Rat80*[a], *D10Mgh6, D10Rat133*[a], *D10Rat151, D10Rat122, D10Rat7* |
| 11 | *D11Mit4, D11Rat21, D11Rat18, D11Rat10, D11Mgh4, D11Mgh3, D11Rat43* |
| 12 | *D12Rat58, D12Rat40, D12Rat39, D12Rat23, D12Rat15, D12Rat52* |
| 13 | *D13Rat2, D13Mgh4, D13Rat25, D13Mit2, D13Mit5, D13Mit4* |
| 14 | *D14Rat1, D14Mit2, D14Rat36, D14Rat16, D14Mit4, D14Rat40, D14Rat57, D14Rat94, D14Rat38, D14Rat132* |
| 15 | *D15Rat69, D15Mgh7, D15Rat5, D15Rat85, D15Mgh8, D15Mgh4, D15Mgh9, D15Rat26, D15Mgh5, D15Rat29* |
| 16 | *D16Mgh4, D16Rat9, D16Rat67, D16Rat64, D16Rat34, D16Rat37* |
| 17 | *D17Rat2, D17Rat8, D17Rat14, D17Rat15, D17Mgh5, D17Rat34, D17Rat37, D17Rat130, D17Rat47* |
| 18 | *D18Rat62, D18Mit1, D18Mit8, D18Rat60, D18Rat8, D18Rat77* |
| 19 | *D19Rat28, D19Rat17, D19Rat15, D19Rat73, D19Mit9, D19Rat68, D19Rat7, D19Rat60* |
| 20 | *D20Rat32, D20Mgh5, D20Rat3, D20Rat7, D20Rat39, D20Rat29* |
| X | *DXRat2, DXRat8, DXRat64, DXRat102* |

[a]Additional SSLP markers for fine mapping

Supplemantal Table S2 SSLP markers used for generation of F344-*Ctns*$^{ugl}$ congenic strain

| Chromosome | SSLP Marker |
|---|---|
| 1 | *D1Rat2, D1Rat153, D1Rat403, D1Rat20, D1Rat27, D1Rat29, D1Rat38, D1Mgh19, D1Arb29, D1Rat70, D1Mit34, D1Rat452, D1Rat90* |
| 2 | *D2Rat251, D2Rat6, D2Wox3, D2Mit5, D2Mgh19, D2Rat215, D2Mit7, D2Mgh24, D2Mit11, D2Rat89, D2Rat118, D2Rat103, D2Rat70* |
| 3 | *D3Rat56, D3Rat193, D3Rat46, D3Rat130, D3Rat39, D3Rat222, D3Mgh11, D3Rat157,D3Mit2, D3Mgh10* |
| 4 | *D4Rat142, D4Mgh13, D4Mgh2, D4Mgh16, D4Rat166, D4Rat176, D4Rat80, D4Arb15, D4Rat72* |
| 5 | *D5Rat120, D5Rat126, D5Rat4, D5Rat141, D5Mit4, D5Mgh23, D5Mgh20, D5Mgh8, D5Rat49* |
| 6 | *D6Rat47, D6Rat41, D6Mit9, D6Wox3, D6Mit8, D6Rat160, D6Rat109* |
| 7 | *D7Rat66, D7Rat113, D7Rat31, D7Rat103, D7Rat24, D7Rat42, D7Rat67, D7Rat81, D7Rat1* |
| 8 | *D8Rat58, D8Mit5, D8Rat62, D8Rat16, D8Rat8, D8Rat171* |
| 9 | *D9Rat44, D9Rat132, D9Rat158, D9Rat16, D9Rat11, D9Rat110, D9Rat2* |
| 10 | *D10Rat95, D10Rat198, D10Rat223, D10Mgh8, D10Rat119, D10Rat80, D10Rat133, D10Rat193, D10Rat122, D10Rat2* |
| 11 | *D11Rat87, D11Mit4, D11Rat10, D11Mgh4, D11Mgh3, D11Rat43* |
| 12 | *D12Rat58, D12Rat40, D12Rat43, D12Rat16, D12Rat25, D12Rat22* |
| 13 | *D13Rat11, D13Rat1, D13Arb5, D13Rat31, D13Mit4, D13Mit15* |
| 14 | *D14Rat1, D14Wox3, D14Rat83, D14Arb10, D14Rat139, D14Mgh4* |
| 15 | *D15Rat114, D15Rat69, D15Rat85, D15Rat92, D15Mgh8, D15Rat28* |
| 16 | *D16Rat88, D16Rat118, D16Rat65, D16Rat34, D16Rat93* |
| 17 | *D17Rat2, D17Rat59, D17Rat15, D17Rat130, D17Rat65* |
| 18 | *D18Mit12, D18Rat132, D18Rat57, D18Mit8, D18Rat8, D18Rat44* |
| 19 | *D19Rat28, D19Rat15, D19Rat46, D19Rat2* |
| 20 | *D20Rat32, D20Rat34, D20Arb10* |

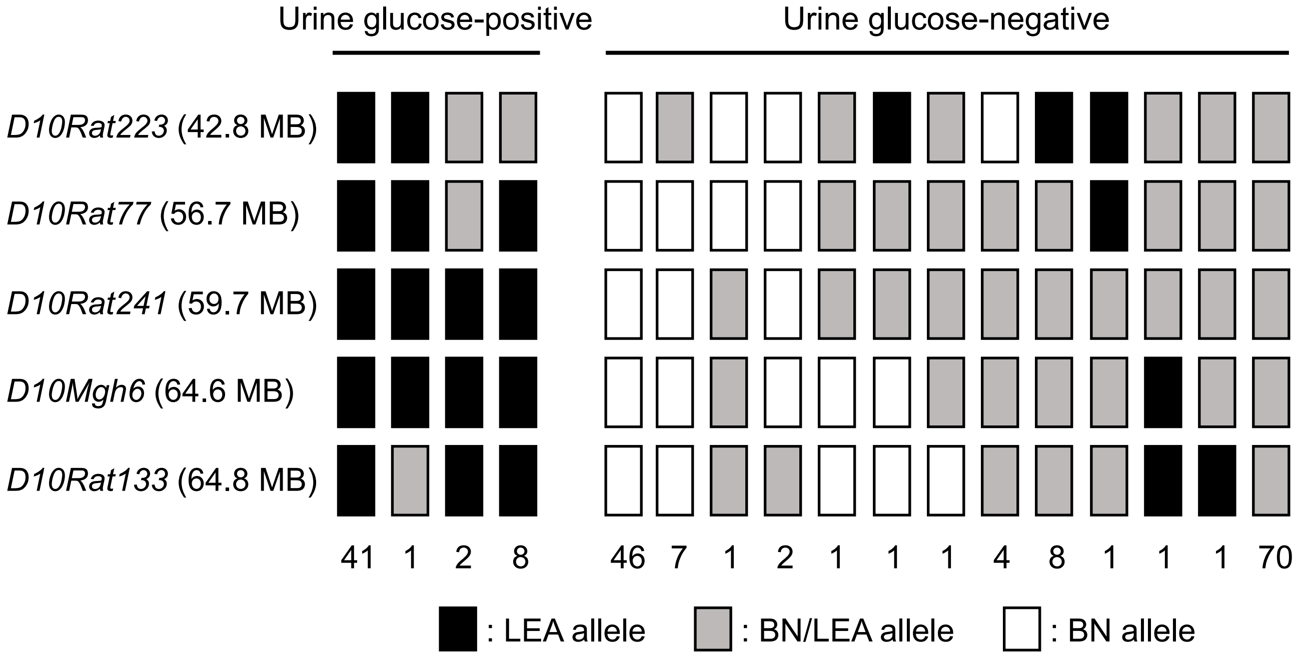


**Supplemental Fig. S1** Linkage analysis of the *ugl* locus. Haplotype analysis of the male 196 $F_2$ intercross progeny. The numbers of offspring that inherited each type of chromosome are listed at the bottom of each column. Position of SSLP markers according to The Rat Genome Database (RGD) are indicated in parentheses. The *ugl* locus was mapped to close to *D10Rat241* on rat chromosome 10.

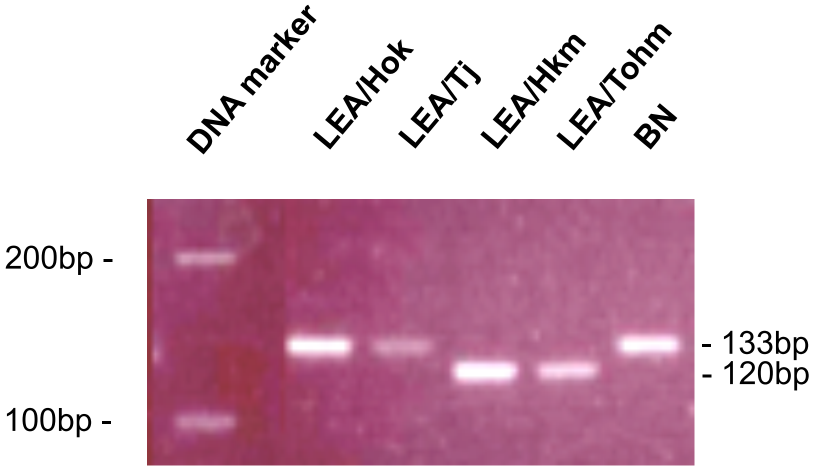


**Supplemental Fig. S2** Variants of the 13-bp deletion in exon 7 of *Ctns* in LEA substrains. Genotyping of the LEA substrains revealed the presence of a 13-bp deletion in LEA/Tohm and LEA/Hkm rats, while it was absent in LEA/Tj and LEA/Hok rats.

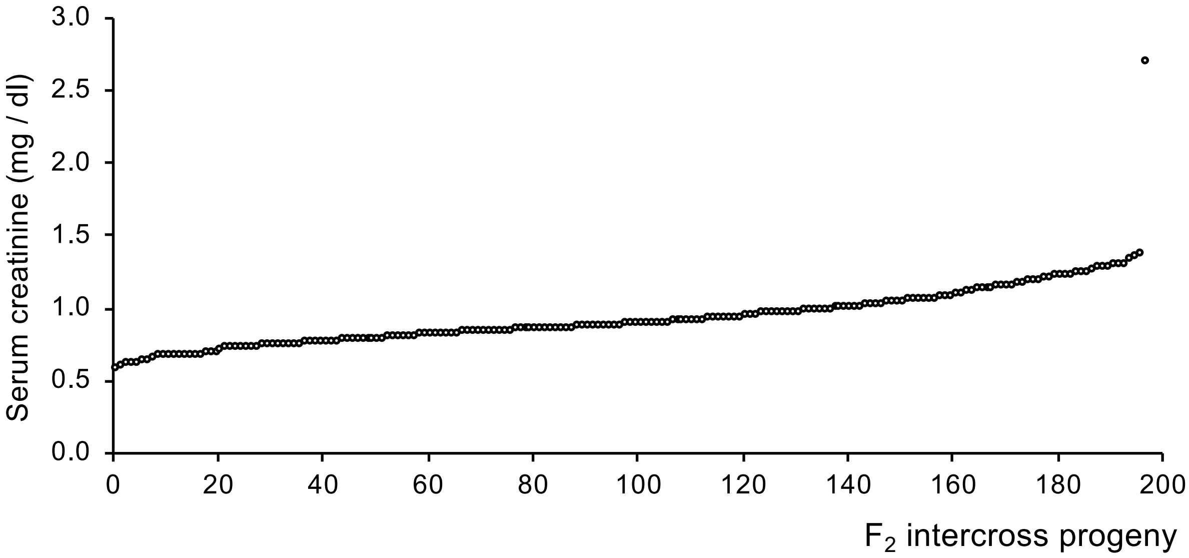


**Supplemental Fig. S3** Distribution of serum creatinine levels in the 196 male $F_2$ intercross progeny.

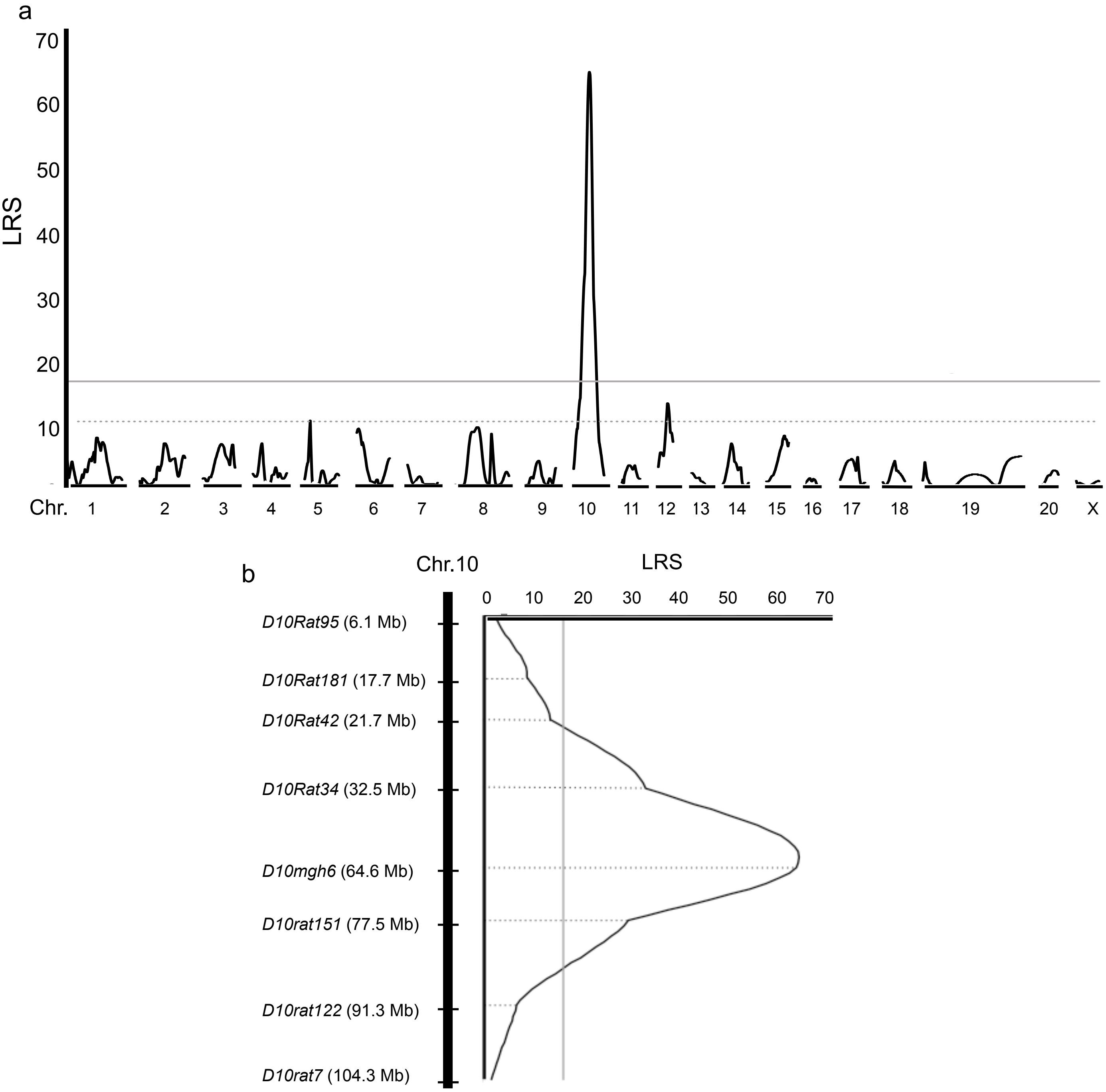


**Supplemental Fig. S4** LRS score plot for QTL of serum creatinine in $F_2$ intercross progeny on whole chromosomes. **(a)** One significant peak (LRS score, 64.1) and one suggestive peak (LRS score, 12.8) are located on chromosome 10 at *D10Mgh6* (64.6 Mb) and 12 at *D12Rat23* (27.2 Mb) respectively. **(b)** Genetic map of chromosome 10. Position of SSLP markers according to The Rat Genome Database (RGD) are indicated in parentheses. The horizontal solid line (LRS score 16.2) and dotted line (LRS score 10.0) indicate significant and suggestive threshold levels of the LRS score, respectively. The thresholds were determined by permutation test.

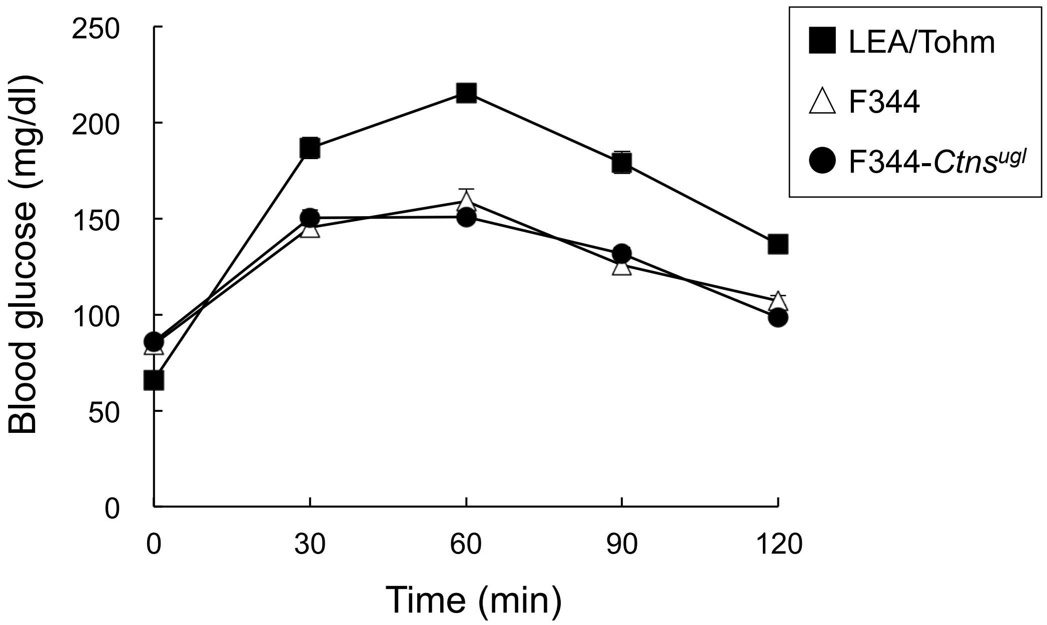


**Supplemental Fig. S5** Oral glucose tolerance test (OGTT) for F344-*Ctns*$^{ugl}$ rats. The glucose tolerance was evaluated by OGTT in male F344 (n = 4), F344-*Ctns*$^{ugl}$ (n = 4) at 10 weeks of age and LEA/Tohm rats (n = 17) at 12 weeks of age. Blood glucose was measured at 0, 30, 60, 90 and 120 min after oral glucose administration.
